# Design of an Anti-HMGB1 Synthetic Antibody for *In Vivo* Ischemic/Reperfusion Injury Therapy

**DOI:** 10.1021/jacs.3c06799

**Published:** 2023-10-16

**Authors:** Hiroyuki Koide, Chiaki Kiyokawa, Anna Okishima, Kaito Saito, Keiichi Yoshimatsu, Tatsuya Fukuta, Yu Hoshino, Tomohiro Asai, Yuri Nishimura, Yoshiko Miura, Naoto Oku, Kenneth J. Shea

**Affiliations:** † Department of Medical Biochemistry, School of Pharmaceutical Sciences, University of Shizuoka, 52-1 Yada, Suruga-ku, Shizuoka 422-8526, Japan; ‡ Department of Chemistry, Missouri State University, 901 South National Avenue, Springfield, Missouri 65897, United States; § Department of Chemical Engineering, Kyushu University, 744 Motooka, Fukuoka 819-0395, Japan; ∥ Department of Chemistry, University of California Irvine, Irvine, California 92697, United States

## Abstract

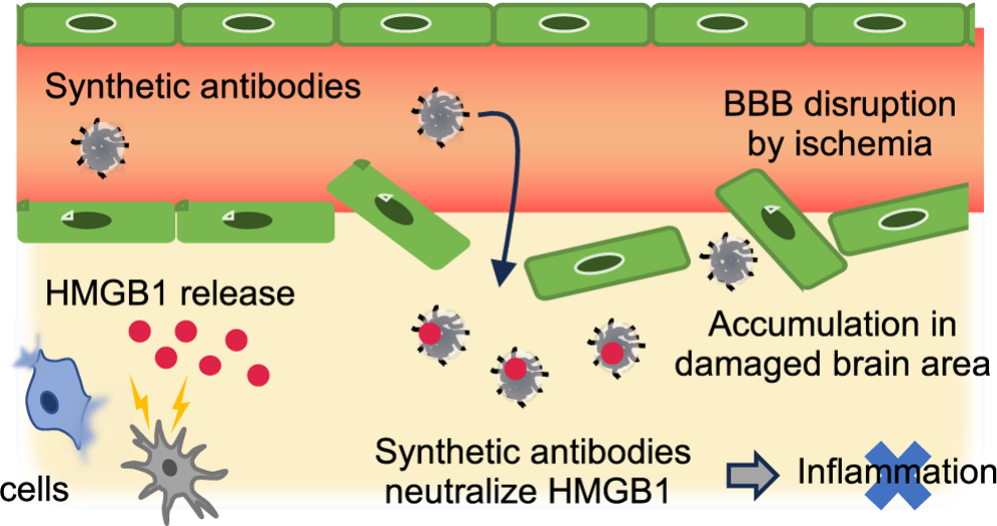

High-mobility group box 1 (HMGB1) is a multifunctional protein. Upon injury or infection, HMGB1 is passively released from necrotic and activated dendritic cells and macrophages, where it functions as a cytokine, acting as a ligand for RAGE, a major receptor of innate immunity stimulating inflammation responses including the pathogenesis of cerebral ischemia/reperfusion (I/R) injury. Blocking the HMGB1/RAGE axis offers a therapeutic approach to treating these inflammatory conditions. Here, we describe a synthetic antibody (**SA**), a copolymer nanoparticle (NP) that binds HMGB1. A lightly cross-linked *N*-isopropylacrylamide (NIPAm) hydrogel copolymer with nanomolar affinity for HMGB1 was selected from a small library containing trisulfated 3,4,6S-GlcNAc and hydrophobic *N*-*tert*-butylacrylamide (TBAm) monomers. Competition binding experiments with heparin established that the dominant interaction between **SA** and HMGB1 occurs at the heparin-binding domain. *In vitro* studies established that anti-HMGB1-**SA** inhibits HMGB1-dependent ICAM-1 expression and ERK phosphorylation of HUVECs, confirming that **SA** binding to HMGB1 inhibits the proteins’ interaction with the RAGE receptor. Using temporary middle cerebral artery occlusion (t-MCAO) model rats, anti-HMGB1-**SA** was found to accumulate in the ischemic brain by crossing the blood–brain barrier. Significantly, administration of anti-HMGB1-**SA** to t-MCAO rats dramatically reduced brain damage caused by cerebral ischemia/reperfusion. These results establish that a statistical copolymer, selected from a small library of candidates synthesized using an “informed” selection of functional monomers, can yield a functional synthetic antibody. The knowledge gained from these experiments can facilitate the discovery, design, and development of a new category of drug.

## 1 Introduction

Protein affinity reagents (PARs) provide molecular recognition for basic research, regulatory testing, biotechnology, diagnostics, and treatment of illnesses such as immune diseases and cancer. Antibodies and their fragments are the best characterized and most widely used PARs; their performance sets the standard for the field. However, despite their essential role, they are not without limitations and shortcomings.^1^ These limitations and the expanding requirements of biotechnology have produced a need for alternatives. The majority are biologicals that include protein platforms such as affimers^2^ and nanobodies^3^ in addition to nonprotein ligands, including oligonucleotide aptamers,^4,5^ peptides,^6,7^ and peptoids.^8^ These materials play an increasingly important role in biotechnology.

Because of this growing need, there is now considerable interest in an emerging class of *abiotic* PARs. Synthetic antibodies (SAs), hydrogel organic copolymers, have been designed with antibody-like affinity for proteins and peptides.^9,10^ Abiotic SAs are carbon backbone copolymers synthesized by a free radical polymerization reaction; their production does not involve living organisms. Libraries of candidates are rapidly synthesized and readily integrated into high-throughput screens. The chemical composition and morphology of SAs differ significantly from their biological counterparts, so they have the potential to offer replacements or in some cases superior alternatives, such as the speed to develop candidate libraries for high-throughput screening, thermal stability, low purification costs, and biological and mechanical robustness over their biological counterparts. There are other strategies, such as molecular imprinting, that are being developed to create synthetic polymers with peptide and protein affinity. This approach differs from that taken in the synthetic antibody approach by including the biomacromolecule target or epitope from the target in the polymerization reaction. The imprint or epitope is then removed from the resulting polymer to reveal a binding site for the target. Several recent notable examples of this approach are given.^11,12^

An interesting difference between biological and synthetic antibodies is in their information content. Protein antibodies are high information content materials; the information resides in their amino acid sequence. Antibody–antigen binding is understood from X-ray crystal structures,^13^ site-directed mutagenesis,^14^ and in silico studies of antibody–antigen complexes.^15^ SAs on the other hand are low information content materials. They are synthesized by a free radical polymerization reaction that produces polymers with a statistical distribution of monomers. The polymers are inhomogeneous materials that lack sequence specificity. Although there is no fundamental reason why synthetic copolymers cannot achieve both protein affinity and selectivity, their inhomogeneity may seem contrary to the traditional understanding of molecular recognition.^16,17^ Nevertheless, SAs have been developed with low nanomolar affinity and high selectivity for protein targets.^18^ They have demonstrated potential for applications for protein isolation^19^ and sensing,^20^ protein separation,^20^ and therapeutic intervention for snake envenomation,^21^ cancer,^18,22^ and sepsis.^23^

In this report, we describe the discovery and *in vitro* and *in vivo* performance of an anti-high-mobility group box 1 (anti-HMGB1) synthetic antibody (SA). HMGB1 (25 kDa, pI = 5.6) is a ubiquitous DNA-binding protein consisting of three distinct segments, an A-box segment (amino acids 1-79) and B-box segment (amino acids 89-162) and an acidic tail (amino acids 186-215).^24^ In addition to its nuclear role, upon injury or inflammation, HMGB1 is released into the extracellular space. Extracellular HMGB1 activates immune and inflammatory responses^25^ by functioning as a crucial cytokine that mediates the response to infection, injury, and inflammation.^24^ Importantly, studies have shown that HMGB1 is involved in the pathogenesis of ischemic stroke and reperfusion injury.^26−31^

**Scheme 1:**
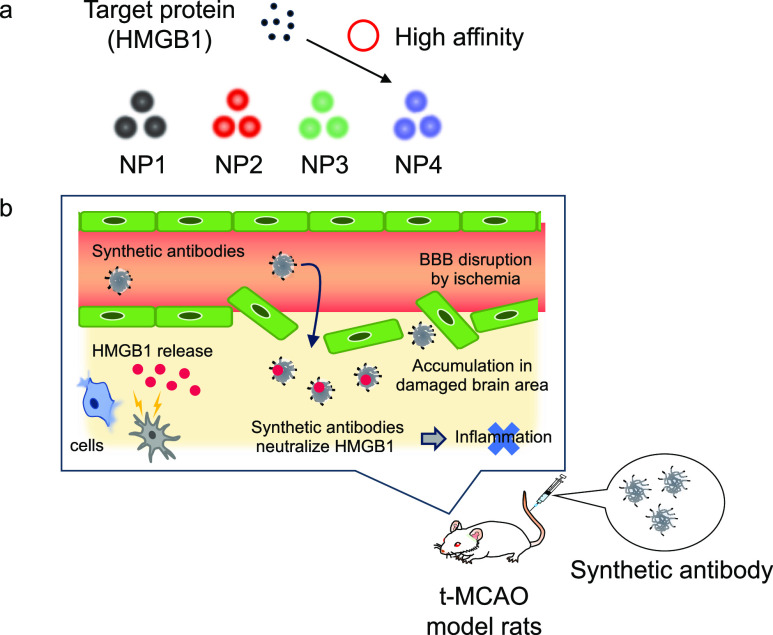
Schematic Strategy of This Study Screening of NPs having high HMGB1 affinity. Ischemic/reperfusion therapy using a synthetic antibody.

This work establishes that an anti-HMGB1-SA can be designed to target a specific domain of a complex protein. Binding is not dominated by nonspecific, intermolecular interactions, but rather the interaction occurs predominately at a local protein domain. The study also provides insight into the distribution of the anti-HMGB1-SA in a living organism and establishes that the SA alters HMGB1 function by inhibiting protein binding to the RAGE receptor *in vitro*. Furthermore, we confirm that the anti-HMGB1-SA passes the blood–brain barrier in ischemic/reperfusion model rats and suppresses the immune and inflammatory responses of cerebral ischemia/reperfusion injury *in vivo* (Scheme 1).

## 2 Results and Discussion

### 2.1 Library Synthesis and Selection of an Anti-HMGB1-SA

**Figure 1:**
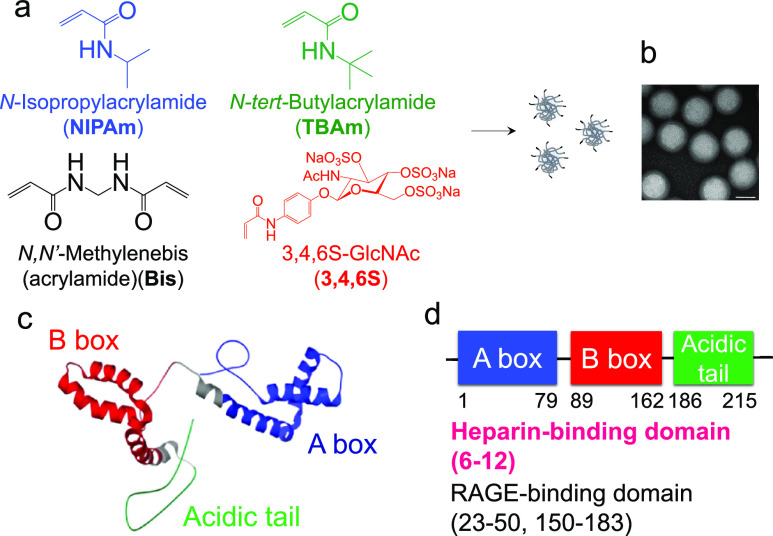
Functional monomers used for nanoparticle synthesis and the HMGB1 structure. (a) Schematic of nanoparticle synthesis. Nanoparticles were synthesized by modified precipitation polymerization in the presence of SDS (0.694 mM) in water using varying amounts of the indicated monomers. The polymerization was initiated by APS addition at 65 °C for 3 h. (b) TEM image of polymer NPs. TEM image of NP3. Scale bar: 50 nm. (c, d) HMGB1 structure.

**Figure 2:**
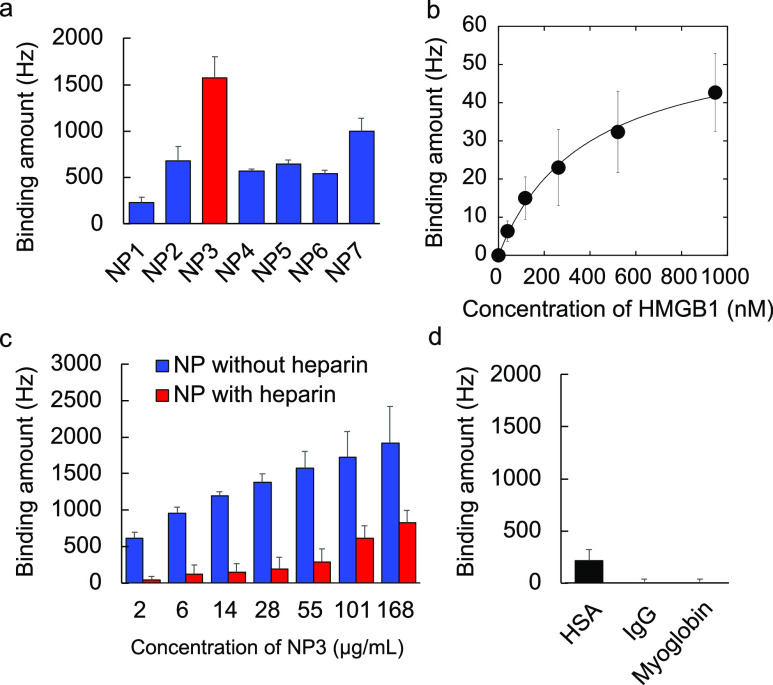
Optimization of the functional monomer percentage in NPs for high affinity to HMGB1. Quartz crystal microbalance (QCM) analysis of HMGB1 or serum protein–NP interaction. The surface of the QCM was functionalized with HMGB1 or serum proteins, and solutions of NPs were added to the QCM cells. (a) HMGB1 affinity for NPs containing 0% (**NP1**), 1% (**NP2**), 1.7% (**NP3**), 3% (**NP4**) 5% (**NP5**), or 10% (**NP6**) 3,4,6S-GlcNAc monomer or 20%TBAm (**NP7**). (b) Affinity of HMGB1 to **NP3**. The surface of the QCM was functionalized with **NP3**. After washing with PBS, HMGB1 was added into **NP3**-immobilized QCM cells to demonstrate the binding affinity of HMGB1 to NP3. (c) HMGB1 affinity for **NP3** in the presence of excess heparin. (d) Serum protein (HSA, myoglobin, or IgG) affinity for **NP3**. Error bars show SD.

Binding and inhibition of HMGB1 function *in vivo* must take into consideration the complexity of the protein target and the unique properties of the synthetic antibody. Prior results from antibody–antigen interactions are not always instructive for predicting the biological behavior of SA–protein complexes. HMGB1 has multiple binding domains that include a heparin-binding domain on Box A, a toll-like receptor (TLR)-binding domain on Box B, and a receptor for advanced glycation end-products (RAGE), also on Box B.^32^ The SA used in this study is substantially larger than that of the protein target. However, affinity alone may not be sufficient to inhibit function. In contrast to globular proteins such as antibodies that interact with antigens at their surface, a lightly cross-linked, low-density hydrogel SA in this study has a porous morphology that can accommodate antigens in their interior. A basic understanding of how these materials inhibit protein function is still lacking. We speculated that direct targeting of one of the above binding domains offered the greatest potential for inhibiting HMGB1 function. Based on prior experience with an anti-VEGF SA,^18^ we chose to target the heparin-binding domain on Box A of HMGB1.^33^ The domain contains a cluster of positively charged arginine residues that reside in the proximity of the RAGE-binding domain. A small library of synthetic polymer nanoparticles (NPs) was prepared by a modified precipitation polymerization of varying compositions and amounts of monomers that included negatively charged monomers containing a trisulfonated *N*-acetylglucosamine (3,4,6S-GlcNAc),^34^*N*-isopropylacrylamide (NIPAm), *N*-*tert*-butylacrylamide (TBAm, a hydrophobic monomer), and 2% *N*,*N*′-methylenebis(acrylamide) (Bis, a cross-linker) (Figure 1). Inclusion of the hydrophobic TBAm was based upon previous studies of protein–NP affinity,^35,36^ which revealed that a combination of electrostatic and hydrophobic interactions increase affinity for the target. Summaries of monomer compositions, particle sizes, and ζ potentials are given in Table 1. The copolymers were purified by dialysis. NP sizes ranged from 60 to 80 nm all with ζ potentials spanning a range of −9 to −17 mV. A transmission electron microscopy (TEM) image confirms the monodispersity of **NP3** (Figure 1). To identify NPs with high affinity to HMGB1, a quartz crystal microbalance (QCM) sensor-immobilized with HMGB1 was used to screen the candidates. The sensor was blocked with bovine serum albumin (BSA, pI = 5.4) to inhibit nonspecific interactions to the QCM gold surface. Interestingly, although HMGB1 is a negatively charged protein (pI = 5.6) at physiological pH, negatively charged **NP3** (1.7% 3,4,6S and 40% TBAm, ζ potential −9 mV) showed the highest affinity to HMGB1 (Figure 2a). The affinity of the NPs for HMGB1 decreased by either increasing or decreasing the % of 3,4,6S-GlcNAc from 1.7 (**NP3**) to 1 (**NP2**) or 3% (**NP4**) compared to **NP1** (0% 3,4,6S). These results demonstrate that the copolymer affinity to HMGB1 is sensitive to small changes in the amount of 3,4,6S-GlcNAc. The falloff in affinity as the 3,4,6S-GlcNAc percentage is increased suggests that NP affinity is responsive to the local composition of the protein surface. Protein binding also decreased by reducing the TBAm percentage from 40% (**NP3**) to 20% (**NP7**) (Figure 2a), indicating the importance of hydrophobic interactions. The affinity of **NP3** for HMGB1 (*K*_d_) was determined by immobilizing **NP3** on the QCM sensor cell and then additional HMGB1 was added to the cell (Figure 2b). The affinity was estimated to be ∼383 nM.^37^ It is noteworthy that variation of just two functional monomers, 3,4,6S-GlcNAc and TBAm, was used to optimize a statistical copolymer NP with high affinity for HMGB1.

**Table 1:** Monomer Composition, Size, PDI, and ζ Potential of the NPs

	**NIPAm**	**TBAm**	**3,4,6S**	**Bis**	**size (d.nm)**	ζ **potential (mV)**	**PDI**
**NP1**	58	40	0	2	60 ± 3	–16 ± 4	0.13 ± 0.07
**NP2**	57	40	1	2	62 ± 3	–17 ± 1	0.17 ± 0.07
**NP3**	56.3	40	1.7	2	56 ± 8	–9 ± 3	0.06 ± 0.02
**NP4**	55	40	3	2	66 ± 12	–13 ± 1	0.2 ± 0.02
**NP5**	53	40	5	2	75 ± 8	–13 ± 6	0.2 ± 0.07
**NP6**	48	40	10	2	85 ± 37	–10 ± 4	0.26 ± 0.03
**NP7**	56.3	20	1.7	2	151 ± 4	–11 ± 5	0.20 ± 0.04

	**NIPAm**	**TBAm**	**AAc**	**Bis**	**size (d.nm)**	ζ **potential (mV)**	**PDI**
**NP8**	56.3	40	1.7	2	79 ± 14	–14 ± 2	0.03 ± 0.03
**NP9**	53	40	5	2	79 ± 4	–15 ± 3	0.09 ± 0.03
**NP10**	48	40	10	2	84 ± 9	–10 ± 2	0.02 ± 0.01

### 2.2 Identifying the SA-HMGB1-Binding Domain

Although the NP composition was formulated with the intention of targeting the heparin-binding domain of HMGB1, the protein presents a complex surface for interaction. To establish the locus of interaction between **NP3** and HMGB1, **NP3** was added to HMGB1-immobilized QCM cells after blocking the heparin-binding domain of HMGB1 by the addition of heparin to the HMGB1-immobilized sensor cells until frequency saturation. Affinity of **NP3** for HMGB1 was dramatically decreased at an **NP3** concentration of ∼50 μg/mL by heparin pretreatment (Figure 2c). The result establishes that there is a significant interaction between **NP3** and the heparin-binding domain of HMGB1. At higher concentrations of **NP3** (above 100 μg/mL), an increase in the frequency (Δ*F*) was noted. We interpret this result by suggesting that at these high **NP3** concentrations, the NP competes for heparin bound to HMGB1 allowing for additional **NP3** binding to the immobilized HMGB1.

We next evaluated the affinity of **NP3** for common serum proteins that span a range of pIs but lack a heparin-binding domain. Human serum albumin (HSA, pI = 4.7), immunoglobulin G (IgG, pI = 6.4), and myoglobin (pI = 7.0) had little affinity to **NP3** (Figure 2d). These results establish that **NP3** binds to HMGB1 at the heparin-binding domain (Box A) of HMGB1. Although statistical copolymers prepared by free radical polymerization lack the sequence specificity of biological antibodies, these results demonstrate that screening a relatively small library of synthetic copolymers using “informed” choices of monomers, complementary to the targeted domain of the protein, can lead to SAs that bind a specific protein domain. Although other strategies, such as molecular imprinting, have also been used to create polymers with peptide and protein affinity, these require including the biomacromolecule target or epitope from the target in the polymerization reaction.^38^

### 2.3 Importance of the Trisulfonated GlcNAc Monomer for HMGB1 Binding

**Figure 3:**
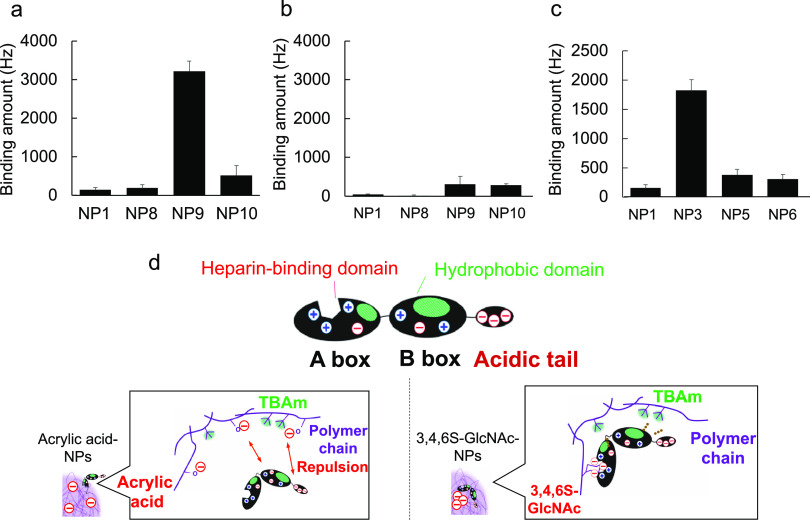
Affinity of NPs for the heparin-binding domain of HMGB1. Quartz crystal microbalance (QCM) analysis of the (a, c) HMGB1 A-box or (b) HMGB1 (whole sequence)–NP interaction. The surface of the QCM was functionalized with HMGB1 A-box or HMGB1 and solutions of NPs were added to the QCM cells. (a) HMGB1 A-box or (b) HMGB1 affinity of 0% (**NP1**), 1.7% (**NP8**), 5% (**NP9**), or 10% (**NP10**) AAc-containing NPs. (c) HMGB1 A-box affinity of 0% (**NP1**), 1.7% (**NP3**), 5% (**NP5**), or 10% (**NP6**) 3,4,6S-containing NPs. Error bars show SD. (d) Schematic image of 3,4,6S or AAc-containing NPs and HMGB1 interaction.

The heparin-binding domain of HMGB1 contains a cluster of three arginines on the A-box of the protein. Competition binding studies established that the locus of interaction between **SA (NP3)** and HMGB1 is at the heparin-binding domain.^30^ The 3,4,6S-GlcNAc monomer presents a tight cluster of three negative charges. Its importance is supported by the fact that **NP1** (0% 3,4,6 NPs) had low affinity to HMGB1. To establish if it is the charge configuration of the monomer that is critical for binding to the heparin-binding domain, we synthesized copolymers with comparable amounts of negative charge using monocarboxylic acid monomers. Although both acidic groups will be ionized at physiological pH, in contrast to the cluster of negative charge in the 3,4,6S-GlcNAc monomer, the dynamic nature of the hydrogel polymer can result in repulsion and separation of individual carboxylate groups. NPs were synthesized with 3 equiv of acrylic acid (AAc) in place of 3,4,6S-GlcNAc. A truncated HMGB1 protein, consisting of only the HMGB1 A-box (pI = 9.7), and the whole HMGB1 (pI = 5.6) protein, were used in these studies. Both proteins contain the heparin-binding domain. Although both 5% AAc-containing NPs (**NP9**, Figure 3a) and 1.7% 3,4,6S-GlcNAc-containing NP (**NP3**, Figure 3b) had high affinity for the HMGB1 A-box, **NP9** had little affinity for HMGB1 (whole sequence, Figure 3c) but, as was established previously, **NP3** had high affinity for HMGB1 (Figure 2a). The lack of affinity of **NP9** for the HMGB1 A-box suggests that it has little specific affinity for the heparin-binding domain. The negligible affinity of **NP9** for HMGB1 could be understood to be the result of electrostatic repulsion due to the acidic tail (overall pI = 5.6). On the other hand, **NP3**’s high affinity for both HMGB1 A-box and whole HMGB1 results from its specific affinity to the heparin-binding domain. This also confirms that the affinity of **NP3** (ξ-potential −9 mV) for HMGB1 (pI = 5.6) is not dominated by electrostatics alone. The concept is illustrated in Figure 3d. The trisulfonated monomer provides a tool to direct binding to a specific basic domain on a protein surface.

### 2.4 Inhibition of HMGB1 Function by NP3 *In Vitro*

Having established that the dominant interaction between **NP3** and HMGB1 occurs at the heparin-binding domain on Box A, the next task was to evaluate the impact of **NP3** binding to HMGB1 function. In general, HMGB1 is stored in the nucleus and released during tissue injury, infection, and inflammation. In the event, extracellular HMGB1 activates immune and inflammatory responses via the interaction with RAGE and TLRs expressed on the endothelial cell surface.^25^

**Figure 4:**
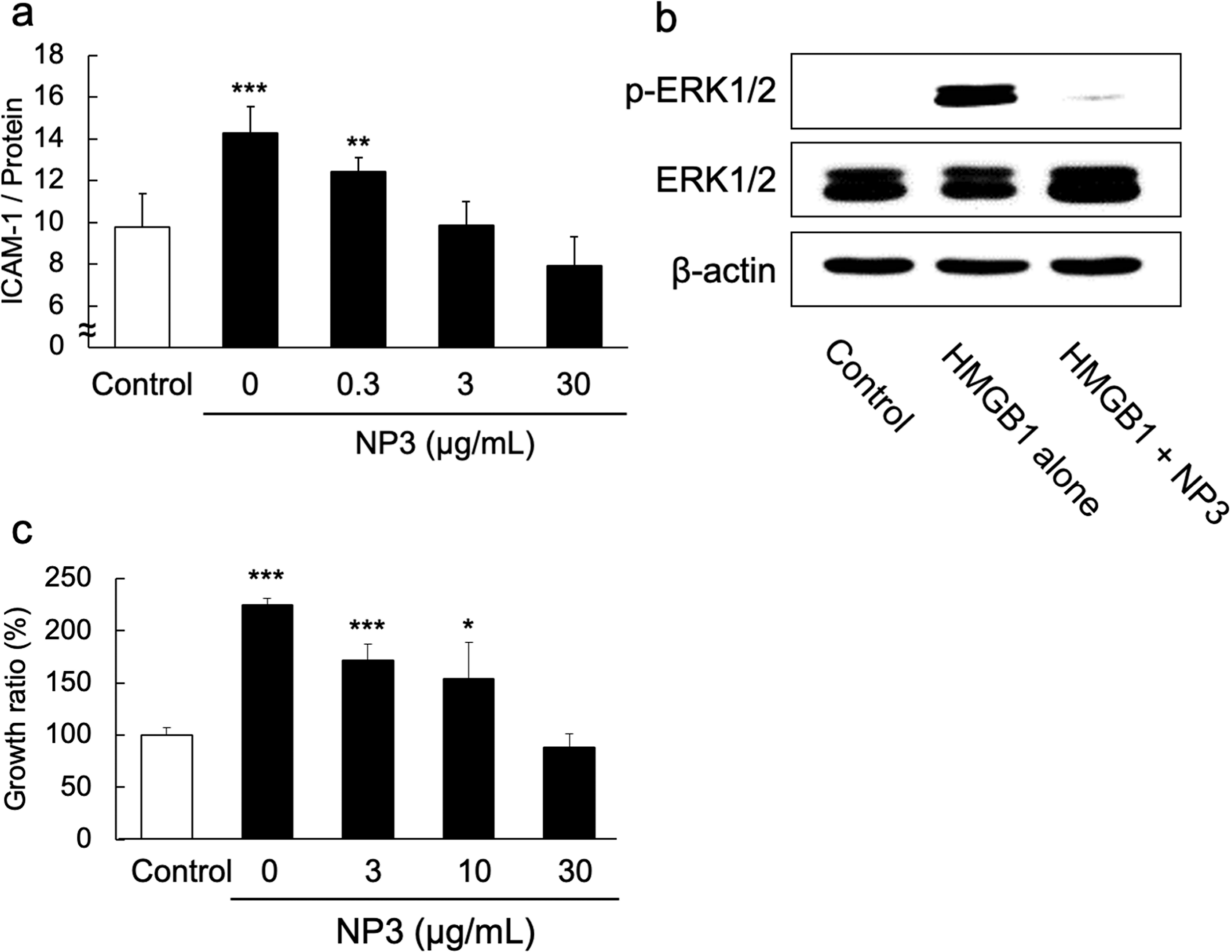
Inhibition of HMGB1 by NP3 *in vitro*. (a) Inhibition of HMGB1-dependent ICAM-1 expression by **NP3**. HUVECs were cultured without growth factors and serum. Twelve hours after the medium change, HUVECs were incubated with HMGB1 (1 μg/mL) and NPs for 16 h at 37 °C. Then, ICAM-1 expression on the cell surface was measured by whole-cell ELISA. Significant differences: * *p* < 0.05 and *** *p* < 0.001 versus 0 μg/mL of **NP3**. (b) HUVECs were cultured without growth factors and serum. Twelve hours after the medium change, cells were incubated with EBM-2 containing 1 μg/mL of HMGB1 and **NP3** (30 μg/mL) for 5 min at 37 °C. Then, phosphorylated ERK1/2 and normal ERK1/2 were detected by Western blotting. The housekeeping gene β-actin was used as a reference. (c) HMGB1 inhibition of RAW264 cell growth. RAW264 cells were incubated with **NP3** (0, 3, 10, and 30 μg/mL) and HMGB1 (3 μg/mL) for 48 h in serum-free medium. The viable cells were determined by WST-8 assay. Data are shown as the mean of viability (%) and SD. Significant difference; *P < 0.05 and *** *P* < 0.001 versus control (without HMGB1).

The RAGE–HMGB1 interaction induces activation of signal transduction pathways in relation to cell motility and proliferation, as well as the production and release of cytokines/chemokines.^25^ It is also known that the RAGE–HMGB1 interaction increases cell surface intercellular adhesion molecule-1 (ICAM-1) and phosphorylated ERK expression. Because of its size (∼56 nm), **NP3** has the potential to restrict access to HMGB1 binding sites on Box B. However, **NP3** is a porous, conformationally dynamic, low-density hydrogel. Its effectiveness in screening protein–protein interactions *in vitro* is still somewhat uncertain. The following experiments were designed to probe the ability of **NP3** to inhibit the production of ICAM-1 and phosphorylated ERK expression through inhibition of the RAGE–HMGB1 interaction *in vitro*. Human umbilical vein endothelial cells (HUVECs) were incubated with HMGB1 (3 μg/mL) and several concentrations of **NP3**. Then, ICAM-1 expression and phosphorylated ERK were measured by whole-cell ELISA or Western blotting.^39^**NP3** dose-dependently inhibited HMGB1-dependent ICAM-1 expression (Figure 4a). In addition, in separate experiments, **NP3** was shown to inhibit ERK phosphorylation at an **NP3** concentration of 30 μg/mL (Figure 4b). Since it is also known that HMGB1 enhances cell growth through interaction with RAGE,^40^ mouse macrophage-like cells (RAW264) were incubated with HMGB1 (3 μg/mL) and/or several concentrations of **NP3**. Then, viable cells were determined by the WST-8 assay. We found that **NP3** dose-dependently inhibited HMGB1-dependent cellular growth (Figure 4c). Collectively, these results indicate that **NP3** binds HMGB1 and inhibits HMGB1-dependent cell downstream signaling cascades via noncovalent inhibition of the HMGB1–RAGE interactions.

As mentioned previously, NP3’s size (∼56 nm) can inhibit protein binding by several mechanisms. Steric screening by the polymer is the most straightforward explanation. However, HMGB1 can also be sequestered in the interior of the low-density, porous nanoparticle.^23^ This also could restrict access to receptor proteins expressed on cell surfaces. At present, we cannot distinguish between these two but ongoing investigations are aimed at understanding in more detail the **NP3**–HMGB1 interaction. Regardless of the details of the inhibition, the results are important for potential therapeutic applications of these SAs. We previously demonstrated the absence of cytotoxicity of NP3 in the range of 0–100 μg/mL.^18^ In addition, NP3 does not induce inflammatory cytokine (TNFα and IL-12) production and body weight change in vivo.^41^

### 2.5 Application of NP3 to Ischemia/Reperfusion (I/R) Injury Treatment

**Figure 5:**
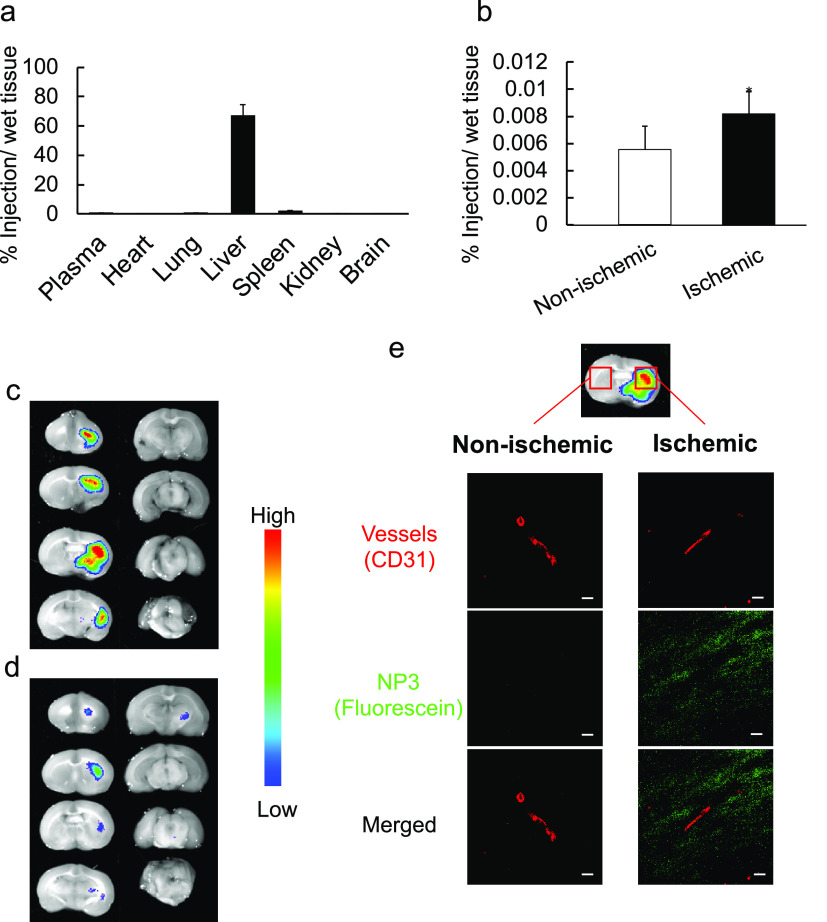
Biodistribution of NP3 in the t-MCAO model rat brain after the intravenous injection. (a, b) Biodistribution of **NP3** in t-MCAO rats. t-MCAO rats were intravenously injected with [^3^H]-labeled **NP3** just after 1 h occlusion. Biodistribution of the **NP3** in the (a) plasma, each organ, and (b) brain at 10 min after the injection was determined by measuring radioactivity. The data show the mean ± SD (*n* = 5), and the significant differences are indicated by brackets: *P < 0.05. (c–e) Localization of **NP3** in the brain of t-MCAO rats. t-MCAO rats were injected with fluorescein-labeled **NP3** at 1 h occlusion. (c) Ten minutes or (d) 6 h later, the brains of the rats were dissected and sliced into 2-mm-thick coronal brain sections. (e) Localization of fluorescein-labeled **NP3** was observed with IVIS. Confocal images of cerebral vessels (CD31, red) and the distribution of fluorescein-labeled **NP3** (green) in the ischemic and nonischemic brain. Scale bar: 20 μm.

Cerebral infarction is caused by a restriction of blood flow to the brain, causing a shortage of oxygen. During ischemic stroke, a cerebral artery occlusion leads to oxygen and nutrient depletion in neural tissues, leading to cell death.^42^ Although reperfusion by thrombolysis treatment is important to suppress extension of the damaged brain area, reperfusion also induces cell death by oxidative stress and inflammatory responses.^43^ This secondary injury is known as a cerebral ischemia/reperfusion (I/R) injury.^43,44^ With recent advances in endovascular therapy including thrombectomy and thrombus disruption, managing reperfusion injury has become an increasingly critical challenge in stroke treatment.^31^ It is known that HMGB1 is a major mediator of I/R injury;^45,46^ therefore, a potential therapeutic strategy is inhibition of extracellular HMGB1 function in the brain to suppress cell death by I/R injury.^27,47^ To exploit this opportunity, the blood–brain barrier (BBB) must be breached. The following experiments were designed to establish **NP3** distribution in transient middle cerebral artery occlusion (t-MCAO) rat models using radiolabeled NPs. These experiments indicated that injected **NP3** accumulated at the site of injury by passing the blood–brain barrier. Higher resolution experiments using fluorescent tagged NPs visualized their localization at the damaged core of the brain, and finally, IV injection of **NP3** enabled evaluation of their therapeutic efficacy. In the event, t-MCAO rats were prepared by introducing a filament into the right MCA to occlude it as a cerebral I/R model.^48^ We first measured the **NP3** biodistribution in the t-MCAO model rats. Radiolabeled **NP3** was prepared by the inclusion of a small amount of [^3^H]-labeled NIPAm into the copolymer to measure the biodistribution of **NP3**. At 1 h occlusion of the t-MCAO model rats, reperfusion was performed by withdrawing the filament. Then, radiolabeled **NP3** was intravenously injected into the t-MCAO model rats immediately after reperfusion (Figure 5a,**b**). Ten minutes after injection of the radioactive particles (**NP3**), plasma and each organ were collected. The brain was separated into ischemic (right brain) and nonischemic (left brain) sections. The radioactivity in each organ was measured. More than 60% of injected **NP3** accumulated in the liver, 30% was in the kidney, and only a few % were in the plasma. Importantly, the accumulation of **NP3** in the ischemic brain was approximately 2 times higher than in the nonischemic brain. In general, hydrogel nanoparticles do not accumulate in the brain following intravenous injection because of the blood–brain barrier (BBB).^49^ However, it was reported that the BBB fails after cerebral ischemia and reperfusion, resulting in lipid nanoparticle accumulation in the brain.^50^ Our results showed that **NP3** also accumulated in the ischemic brain, predominantly at the site of injury in the damaged part of the brain.

To further investigate the localization of **NP3** in the brain of the t-MACO rat, a fluorescein-labeled **NP3** was synthesized by inclusion of a fluorescein monomer (1 mol %) in the polymerization. Fluorescein-labeled **NP3** was intravenously injected into the t-MCAO rats just after reperfusion (Figure 5c,d). Ten minutes after the injection, mice were anesthetized and the brain was collected and sliced into 8 sections to observe the localization of **NP3**. The fluorescein-labeled **NP3** localization in the brain was measured by an *in vivo* imaging system (IVIS). Although no signal was observed in the left (nonischemic) brain, a strong signal was observed in the right (ischemic) brain, especially section nos. 3–5. Since the ischemic core in the t-MCAO rat is also located at the center of section nos. 3–5, the imaging studies established the correspondence between **NP3** and the ischemic core, and **NP3** accumulated in the damaged brain area as a result of the BBB disruption. We next observed the intrabrain distribution of **NP3** in the sliced brain section no. 4 of both the right (ischemic) and left (nonischemic) brain using confocal laser scanning microscopy (Figure 5d). There were no fluorescein (NP) signals in proximity to the blood vessels in the left (nonischemic) brain. However, a fluorescein signal of **NP3** was observed in the proximity of blood vessels in the right (ischemic) brain. To summarize, **NP3** leaks from damaged blood vessels and accumulates in the brain. From these biodistribution results, IV injected **NP3** has the potential to capture and inhibit HMGB1 following reperfusion in the ischemically damaged brain.

**Figure 6:**
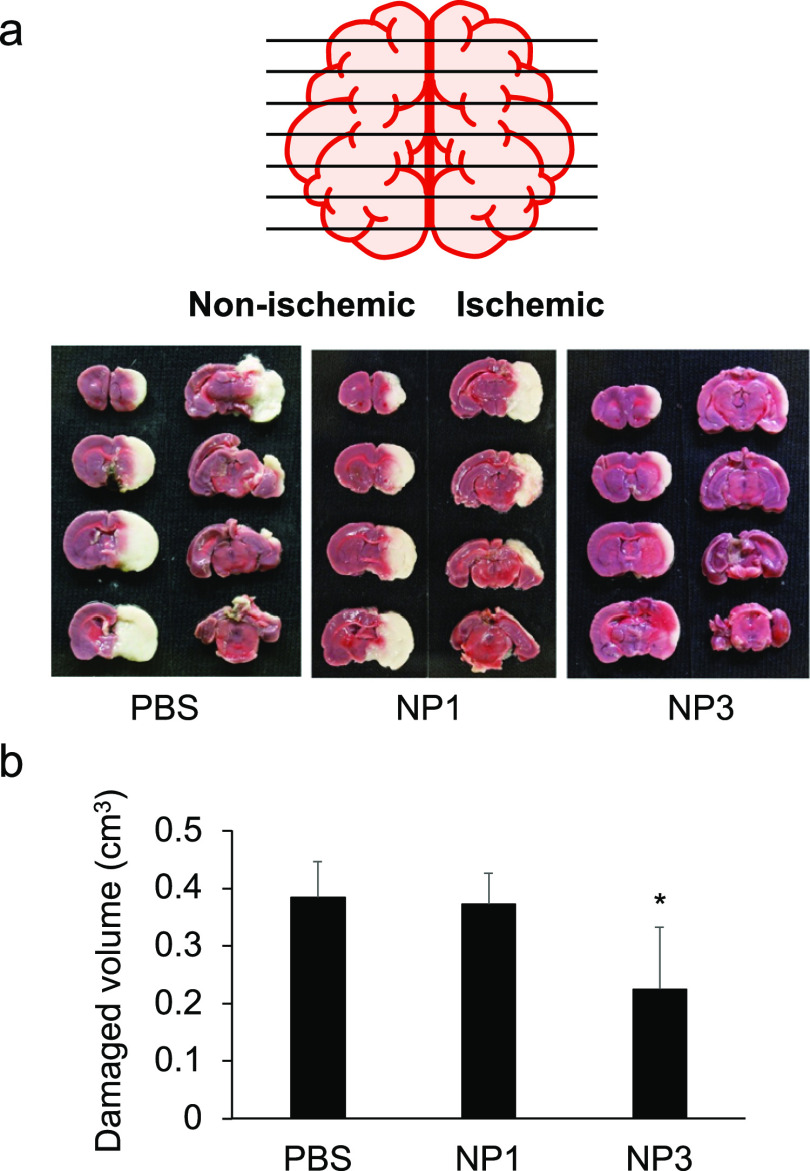
Therapeutic effect of NP3 against cerebral ischemia/reperfusion injury. (a) t-MCAO rats were intravenously injected with PBS, **NP1** (control, 10 mg/kg), or **NP3** (10 mg/kg) just after 1 h occlusion. At 1 h after injection, the brains of the rats were reperfused. (a) Twenty-four hours after the occlusion, 2 mm coronal brain sections were stained with TTC for 30 min at 37 °C. (b) Damaged brain volume (white area) was calculated by using ImageJ. Data are presented as the mean ± SD (*n* = 5). Significance of differences: *P < 0.05.

The therapeutic effect of **NP3** on I/R injury was determined using the t-MCAO rat. PBS, **NP3**, or **NP1** (control NP, 0% 3,4,6S, and 40% TBAm) was intravenously injected at 0 and 6 h after reperfusion (10 mg/kg). Twenty-four hours after reperfusion, the brain was sliced into 8 sections and stained with 2,3,5-triphenyltetrazolium chlorides (TTCs) to detect damaged brain area (Figure 6). Damaged brain volume (white area) was not significantly different between PBS and **NP1** treatment. However, the damaged brain area was significantly reduced following injection of **NP3**. These results are attributed to neutralization of HMGB1 in the damaged ischemic area. The intervention resulted in a significant decrease in the damaged brain area through suppression of inflammation. **Anti-HMGB1-SA-NP3** is demonstrated to have therapeutic efficacy for treatment of cerebral ischemia/reperfusion injury in t-MCAO rats.

## 3 Conclusions

A small library of 2% cross-linked NIPAm synthetic copolymers was prepared from trisulfonated 3,4,6S-GlcNAc and hydrophobic TBAm monomers. A copolymer nanoparticle with a nanomolar affinity for HMGB1, a synthetic antibody (**anti-HMGB1-SA**), was selected from the library. Competition binding experiments with heparin established that the dominant interaction of **anti-HMGB1-SA** and HMGB1 occurred at the heparin-binding domain of HMGB1 (Box A). *In vitro* studies established that **anti-HMGB1-SA** inhibits HMGB1-dependent ICAM-1 expression and ERK phosphorylation of HUVECs establishing that **anti-HMGB1-SA** inhibits the biological function of HMGB1. As the HMGB1–RAGE interaction contributes importantly to stroke-induced cerebral ischemic/reperfusion injury, blocking that interaction offers a pathway to reduce I/R injury. Therapeutic intervention requires passage of the blood–brain barrier. In the event following reperfusion, ^3^H-labeled **anti-HMGB1-SA** was found to accumulate in the brain presumably by passage through damaged blood vessels, offering the potential to inhibit HMGB1 in the I/R damaged brain. Subsequent experiments involving intravenous injection of **anti-HMGB1-SA** to t-MCAO rats dramatically reduced brain damage induced by cerebral ischemia/reperfusion. The results establish that **SA** can be identified by a selection process from a small library of copolymers formulated with monomers bearing functional groups complementary to the biological target. The selected copolymer exhibits nanomolar affinity for a complex, multifunctional protein at a specific domain and inhibits its function *in vitro* and *in vivo*. Although many hurdles must be overcome before therapeutic applications are realized, these results offer a promising abiotic alternative to protein affinity reagents that function in living systems. The knowledge gained in this study advances our understanding of SA–protein binding and can facilitate the discovery, design, and development of a new genre of protein affinity reagents.
