# Supplementary material for: Design of an Anti-HMGB1 Synthetic Antibody for In Vivo Ischemic/Reperfusion Injury Therapy
Source: J Am Chem Soc. 2023 Oct 16;145(42):23143–51. doi: 10.1021/jacs.3c06799 (PMC10603801; doi:10.1021/jacs.3c06799)
Supplement: Supplementary file 1 — ja3c06799_si_001.pdf [file ja3c06799_si_001.pdf]

Design of An Anti-HMGB1 Synthetic Antibody for in vivo Ischemic/Reperfusion Injury Therapy

Hiroyuki Koide[1], Chiaki Kiyokawa[1], Anna Okishima[1], Kaito Saito[1], Keiichi Yoshimatsu[2], Tatsuya Fukuta[1], Yu Hoshino[3], Tomohiro Asai[1], Yuri Nishimura[3], Yoshiko Miura[3], Naoto Oku[1] and Kenneth J. Shea[2]**

[1]Department of Medical Biochemistry, Graduate division of Pharmaceutical Sciences, University of Shizuoka, 52-1 Yada, Suruga-ku, Shizuoka, Shizuoka 422-8526, Japan. [2]Department of Chemistry, University of California Irvine, Irvine, CA 92697 USA., [3]Department of Chemical Engineering, Kyushu University, 744 Motooka, Fukuoka 819-0395, Japan.

*To whom correspondence should be addressed.

Kenneth J. Shea

TEL: 949-824-5844, FAX: 949-824-2210, e-mail: kjshea@uci.edu

Hiroyuki Koide

TEL: +81-54-264-5702, FAX: +81-54-264-5705, e-mail: hkoide@u-shizuoka-ken.ac.jp

Supporting Information

Materials and Methods

Materials and Methods

Materials

N-isopropylacrylamide (NIPAm), bovine serum albumin (BSA), IgG, myoglobin, human serum albumin (HSA), anti-ß actin rabbit monoclonal antibody, sodium dodecyl sulfate (SDS) and fluoresceinamine isomer were purchased from Sigma Aldrich (St Louis, MO, USA). *N*,*N'*-methylenebisacrylamide (BIS) and, *N*-t-butylacrylamide (TBAm) was procured from TCI (Tokyo, Japan). HMGB1 was purchased from R&D systems (Minneapolis, MN, USA). Anti-ERK1/2 rabit monoclonal antibody and anti-phospho-ERK1/2 rabit monoclonal antibody were purchased form the Cell Signaling Technology (Danvers, MA, USA). Biotin conjugate anti-rat CD31 antibody was purchased from BD Pharmingen (San Diego, CA, USA). Avidin conjugate Alexa594 was purchased from Molecular probes (Eugene, OR, USA). HMGB1 box A was purchased from HMGBiotech Srl (Milano, Italy). [^{3}H]*N*-Isopropylacrylamide (3.27 GBq/mg) was purchased from American Radiolabeled Chemicals, Inc. (St Louis, MO, USA)

Preparation of NPs.

NPs were synthesized by the free-radical copolymerization of *N*-isopropylacrylamide (NIPAm) cross-linked with 2 mol% *N*,*N'*-methylenebisacrylamide (Bis)[46]. *N*-t-butylacrylamide (TBAm), 3,4,6S-GlcNAc[30] and acrylic acid (AAc) were used as hydrophobic and negatively charged functional monomers. NIPAm

(98−(W+X+Y+Z) mol%), 3,4,6S (W mol%), AAc (X mol%), TBAm (Y mol%), BIS (2 mol%), and SDS (10 mg) were dissolved in water (50 mL) and the resulting solutions were filtered through a no. 2 Whatman filter paper. TBAm (Z mol%) was dissolved in ethanol (1 mL) before addition to the monomer solution, which resulted in a total monomer concentration of 65 mM. For the preparation of radio-labeled NPs, a small amoun of [^{3}H]-labeled *N*-isopropylacrylamide was added into the solution. In addition, FITC-monomer was added to the initial solution (1 mol%) for the preparation of FITC-labeled NPs. Nitrogen was bubbled through the reaction mixtures for 30 min. Following the addition of ammonium persulfate aqueous solution (30 mg per 500 μL), the polymerization was carried out at 65 °C for 3 h under a nitrogen atmosphere. The polymerized solutions were purified by dialysis against an excess of pure water (changed more than twice a day) for >4 days.

Characterization of NPs

The hydrodynamic diameter of NPs was determined in 1 mM phosphate buffer (pH7.4) by dynamic light scattering (DLS) at 25 ± 0.1 °C (Zetasizer Nano ZS).

Transmission electron microscopy image

NPs (1 mg/ml) in a volume of 5 μL were placed on a grid (Nisshin EM, Tokyo, Japan) and dried by a stream of warm air 3 times. Then, each sample was negatively stained with 10 μL of 1 w/v% ammonium molybdate for 1 min and imaged with an HT7700 TEM System (Hitachi High-Technologies, Tokyo, Japan). The images

were recorded with a CCD camera at 1024 x 1024 pixels (Advanced Microscopy Techniques, Woburn, MA, USA).

Quartz crystal microbalance (QCM) analysis

An Affinix Q4 and Q8 QCM instruments (Alvac Co. Ltd., Kanagawa, Japan) were used to quantify the interactions between the NPs and proteins. At first, gold electrodes were cleaned with piranha solution for 5 min, twice. 3,3'-Dithiodipropionic acid (1 mM, 0.1 mL) was added into the QCM cells and incubated overnight. Then, the QCM cells were washed with pure water and carboxylic acids on the electrodes were activated by loading 1-ethyl-3-(3-dimethylaminopropyl)-carbodiimide (100 mg/ml) and *N*-hydroxysuccinimide (100 mg/mL) (1:1) aqueous solution (0.1 mL) to form *N*-hydroxysuccinimidyl esters. For immobilization of HMGB1, the proteins (30 μg/mL) diluted by nano pure water was added onto the QCM gold surface (10 μL) and incubated for 3 h at 37°C. Then, the QCM cells were washed with pure water twice, and blocked with 1 mg/mL BSA solution for 1 h. Following washing, NPs were added into the cells at concentrations of 1.99, 5.9, 13.6, 28.3, 55.2, 100.6, 168.43 μg/ml. Interactions between NPs and proteins were observed at (37±0.1)°C in PBS (pH 7.4). The apparent dissociation constant of NPs to protein was calculated under the assumption that all particles have the same affinity to protein.

Apparent equilibrium dissociation constant (K_d)

An Affinix Q4 QCM instrument (Alvac Co. Ltd., Kanagawa, Japan) was used to quantify the interactions between the NPs and HMGB1. HMGB1 was immobilized on the QCM cells by amino coupling as shown in QCM analysis. NPs were added to the cells until saturation. Then, the QCM cells were washed with PBS twice. HMGB1 was then added into the cell to measure the affinity of NPs for HMGB1 at (37±0.1)°C in PBS (pH 7.4). The apparent dissociation constant of NPs to protein was calculated under the assumption that all particles have the same average affinity to protein[33].

Cell culture

Human umbilical vein endothelial cells (HUVECs, Takara Bio, Otsu, Shiga, Japan) were cultured in endothelial growth medium-2 (EGM-2, Cambrex Corporation, Walkersville, USA) at 37 °C under 5% CO_2 in the air. RAW264 cells (ATCC, Virginia, USA) were cultured in DME/high-glucose medium (Wako Pure Chemical Industries, Ltd., Osaka, Japan) supplemented with 10% FBS, 100 U/mL penicillin, and 100 μg/mL streptomycin at 37°C in a humidified atmosphere of 5% CO_2 in the air.

Phosphorylated ERK1/2

HUVECs were seeded onto 6 well plates at the density of 2×10^5 cells/well and incubated overnight. Then, the culture medium was changed to EBM-2 that does not contain growth factors and serum. Twelve hours after the medium change, cells were incubated with EBM-2 containing 1 μg/mL of HMGB1 and NPs (30 μg/mL) for 5 min at 37 °C. For negative control, cells were incubated with only EBM-2 (no HMGB1 and NPs). The

cells were then washed with PBS and lysed with lysis buffer composed of 10 mM Tris (pH 7.5), 0.1% SDS, 50 µg/mL aprotinin, 200 µM leupeptin, 2 mM PMSF, 100 µM pepstatin A and 1 mM Na_3VO_4. The total protein concentration was measured by using a BCA Protein Assay Reagent Kit (PIERCE Biotechnology, Rockford, IL). The cell extracts were subjected to 7.5% SDS–PAGE and transferred electrophoretically to polyvinylidene difluoride (PVDF) membranes (Millipore, Billerica, MA, USA). After having been blocked for 1 h at room temperature with 5% BSA in Tris–HCl-buffered saline containing 0.1% Tween 20 (TTBS, pH 7.4), the membranes were incubated with a primary antibody (against β-actin, ERK1/2, or phosphor-ERK1/2) for 24 h at 4 °C. Then, they were incubated for 1 h at room temperature with HRP-conjugated secondary antibody at a dilution of 1:2000. Each sample was developed by using a chemiluminescent substrate (ECL; GE Healthcare Bioscience), and each protein was detected with the LAS-3000 mini system.

ICAM-1 expression

HUVECs were seeded onto 96 well plates at the density of 6×10^3 cells/well and incubated overnight. Then, the culture medium was changed to EBM-2 that does not contain growth factors and serum. Twelve hours after the medium change, cells were incubated with EBM-2 containing 1 µg/mL of HMGB1 and different concentration of NPs for 16 h at 37 °C. For negative control, cells were incubated with only EBM-2 (no HMGB1 and NPs). The cells were then incubated with 4% paraformaldehyde for 15 min. After the washing with PBS, the cells were incubated with primary antibody (anti-ICAM1) for 1 h at 37 °C. Then, they were

incubated for 1 h at 37 °C with HRP-conjugated secondary antibody. Each sample was developed by using Tecan Infinite M200 microplate reader.

Cell growth assay

RAW264 were seeded on 96 well plate at the density of 1.0×10^5 with HMGB1 (3 µg/ml) and several concentrations of NPs. Forty-eight hours after the seeding, Cell Counting Kit-8 (Dojindo, Kumamoto, Japan) was added to each well in accordance with the manufacturer's instructions. Then, absorbance was measured with a Tecan Infinite M200 micro plate reader at a test wavelength of 450 nm and a reference wavelength of 630 nm.

Experimental animals

Seven-week-old Wistar male rats were purchased from Japan SLC Inc. (Shizuoka, Japan). The animals were cared for according to the Animal Facility Guidelines of the University of Shizuoka. All animal procedures were approved by the Animal and Ethics Review Committee of the University of Shizuoka.

Preparation of transient middle cerebral artery occlusion (t-MCAO) model rats

Transient middle cerebral artery occlusion (t-MCAO) model rats were prepared as described previously[45,47]. Briefly, rats were induced anesthesia with 3% isoflurane and maintained with 1.5% isoflurane during the surgery (37 °C). After a median incision of the neck skin, the right carotid artery, external carotid artery, and

internal carotid artery (ICA) were isolated with careful conservation of the vagal nerve. A 4-0 monofilament nylon filament coated with silicon was introduced into the right ICA and advanced to the origin of the MCA to occlude it. Silk thread was used for ligation to keep the filament at the site of insertion into the MCA. After the operation, the neck was closed and anesthesia was discontinued. MCAO was performed for 1 h. Success of the surgery was judged by the appearance of hemiparesis. Reperfusion was performed by withdrawing the filament about 10 mm at 1 h after the occlusion under isoflurane anesthesia.

Biodistribution of NP3 in t-MCAO rat

t-MCAO rats were intravenously injected with [^{3}H]-labeled **NP3** just after the reperfusion (112 kBq/rat). At 10 min after the injection, the rats were sacrificed under deep anesthesia with isoflurane for the collection of the blood. Then, the blood was heparinized and separated by centrifugation (700 × g, 15 min, 4°C) to obtain the plasma. Then, their heart, lungs, liver, spleen, kidneys and brain were removed and weighed. The brain was separated into ischemic and non-ischemic section. The radioactivity in plasma and each organ was determined with a liquid scintillation counter (LSC-3100, Aloka, Tokyo, Japan).

Localization of NP3 in the brain

t-MCAO rats were intravenously injected with FITC-labeled **NP3** (6.4 mg/kg) just after the reperfusion. At 10 min after the injection, the brains were dissected and sliced into 2-mm thick coronal sections with a rat brain slicer (Muromachi Kikai, Tokyo, Japan). Then, the fluorescence activities were detected with an *in vivo*

imaging system (IVIS, Xenogen Corp., Alameda, CA). After the imaging, these samples were frozen using dry ice-ethanol. These frozen sections were cut into 10 μm slices using a cryostat (HM505E, Microm, Walldorf, Germany) and mounted with Perma Fluor Aqueous Mounting Medium (Thermo Fisher Scientific Inc., Yokohama, Japan). Then, the slices were blocked with 1% BSA for 10 min at room temperature and stained with biotin-conjugated anti-CD31 antibody for 18 h at 4°C. After the washing with PBS, the slices were stained with avidin-conjugated Alexa594 for 30 min at room temperature. Then, the slices were incubated with 4% PFA for 15 min at room temperature and monitored with confocal laser-scanning microscope (Nikon, Tokyo, Japan).

Therapeutic effect

t-MCAO rats were intravenously injected with PBS, **NP1** (control) or **NP3** (6.4 mg/kg) at 0 and 6 h just after the reperfusion. At 24 h after the reperfusion, the brains were dissected and sliced into 2-mm thick coronal sections. Then, the slices were stained with 2% TTC solution for 30 min at 37 °C to assess the damaged brain area. The damage volume was calculated with an image-analysis system (NIH Image J).
